# Thrombopoietin Receptor Agonists for Thrombocytopenia Secondary to HER2-Targeted Antibody Drug Conjugates

**DOI:** 10.1093/oncolo/oyad185

**Published:** 2023-06-19

**Authors:** Michael Rainone, Saro Kasparian, Tina Nguyen, Neel Talwar, Yuan Yuan, Matthew Mei, Joanne E Mortimer, James R Waisman, Niki Patel, Vinod Pullarkat

**Affiliations:** Department of Medical Oncology and Therapeutics Research, City of Hope Comprehensive Cancer Center, Duarte, CA, USA; Department of Hematology and Hematopoietic Cell Transplantation, City of Hope Comprehensive Cancer Center, Duarte, CA, USA; Department of Medical Oncology and Therapeutics Research, City of Hope Comprehensive Cancer Center, Duarte, CA, USA; Department of Hematology and Hematopoietic Cell Transplantation, City of Hope Comprehensive Cancer Center, Duarte, CA, USA; Department of Hematology and Hematopoietic Cell Transplantation, City of Hope Comprehensive Cancer Center, Duarte, CA, USA; Department of Medical Oncology and Therapeutics Research, City of Hope Comprehensive Cancer Center, Duarte, CA, USA; Department of Medical Oncology and Therapeutics Research, City of Hope Comprehensive Cancer Center, Duarte, CA, USA; Department of Medical Oncology, Cedars-Sinai Medical Center, Los Angeles, CA, USA; Department of Hematology and Hematopoietic Cell Transplantation, City of Hope Comprehensive Cancer Center, Duarte, CA, USA; Department of Medical Oncology and Therapeutics Research, City of Hope Comprehensive Cancer Center, Duarte, CA, USA; Department of Medical Oncology and Therapeutics Research, City of Hope Comprehensive Cancer Center, Duarte, CA, USA; Department of Medical Oncology and Therapeutics Research, City of Hope Comprehensive Cancer Center, Duarte, CA, USA; Department of Medical Oncology, Cedars-Sinai Medical Center, Los Angeles, CA, USA; Department of Hematology and Hematopoietic Cell Transplantation, City of Hope Comprehensive Cancer Center, Duarte, CA, USA

**Keywords:** HER2 positive breast cancer, trastuzumab deruxtecan, trastuzumab emtansine, thrombocytopenia, romiplostim, avatrombopag

## Abstract

Trastuzumab emtansine and trastuzumab deruxtecan are widely used in breast cancer and other solid tumor malignancies. Thrombocytopenia is a common adverse event associated with the use of these agents that can lead to a treatment delay, reduction in dose intensity, and discontinuation. The role of thrombopoietin receptor agonists (TPO-RA) remains unknown in this setting. We report a case series of 6 individuals with breast cancer that experienced dose-reductions and therapy delays due to thrombocytopenia secondary to trastuzumab emtansine or trastuzumab deruxtecan therapy and received intervention with TPO-RA. All 6 were able to resume therapy with TPO-RA support.

## Teaser

Thrombopoietin receptor agonists (TPO-RA), such as eltrombopag, romiplostim, and avatrombopag, have shown efficacy in treating immune thrombocytopenia; however, their efficacy in improving platelet counts due to HER2-targeted antibody-drug conjugate therapy remains unknown. This article reports a series of cases in which TPO-RA were successfully utilized to treat such thrombocytopenia associated with ado-trastuzumab emtansine(T-DM1) and fam-trastuzumab deruxtecan (T-DXd) therapy.

## Introduction

Thrombocytopenia is a common adverse event associated with the HER2-targeted antibody-drug conjugates (ADC) ado-trastuzumab emtansine (T-DM1) and fam-trastuzumab deruxtecan (T-DXd). Thrombocytopenia can lead to treatment delays, reduction in dose intensity and in some cases therapy discontinuation.^1^ T-DM1 and T-DXd are widely used in the treatment of breast cancers with HER2 expression.^2-6^ T-DM1 has received Food and Drug Administration (FDA) approvals for adjuvant HER2 positive breast cancer after prior neoadjuvant therapy with residual cancer and for late-stage HER2 positive breast cancer. T-DXd has FDA approvals for HER2 positive, and HER2 low, unresectable, or metastatic breast cancer after prior chemotherapy, for previously treated HER2 positive gastric cancer, and for previously treated HER2 mutant non-small cell lung cancer. T-DXd has a role in the second-line treatment of metastatic HER2 positive breast cancer and emerged as a treatment option for advanced HER2-low breast cancer which has substantially expanded its role.^4-6^

In key breast cancer trials of these agents, grade ≥ 3 thrombocytopenia was observed in 5.7%-24.9% of individuals treated with T-DM1 and 4.3%-7.0% in those treated with T-DXd.^2-6^ In the DESTINY-Gastric01 study 14 of 125 (11%) participants experienced grade ≥ 3 thrombocytopenia in the T-DXd arm.^7^ In the TH3RESA study 24 of 404 (6%) of participants experienced grade ≥ 3 thrombocytopenia on T-DM1.^8^ The phase 3 KATHERINE trial that established adjuvant T-DM1 as the standard of care for residual cancer at surgery following neoadjuvant chemotherapy plus trastuzumab reported that 31 of 740 (4.2%) participants in the T-DM1 arm discontinued therapy due to thrombocytopenia.^3^

Thrombopoietin receptor agonists (TPO-RA) such as eltrombopag, romiplostim, and avatrombopag have shown efficacy in treating immune thrombocytopenia. Their efficacy in improving platelet counts due to HER2-targeted antibody-drug conjugate (ADC) therapy remains unknown. We report a series of cases in which TPO-RA was successfully utilized to treat such thrombocytopenia associated with T-DM1 and T-DXd therapy.

## Materials and Methods

A retrospective chart review was performed to identify individuals treated with T-DM1 or T-DXd who developed therapy-induced thrombocytopenia that required a treatment delay and/or a reduction in dose intensity at our institution between January 1, 2018, and June 30, 2022, and they are representative of our patient population from Los Angeles, Orange, and San Bernardino counties in California. The individuals had good disease control on their HER2-targeted ADC, and subsequently received treatment with a TPO-RA at the discretion of their medical teams after alternative etiologies for thrombocytopenia were excluded clinically by hematologists. This study was carried out following institutional review board approval and procedures were followed in accordance with the ethical standards of the Helsinki Declaration.

## Results

**Figure 1.**
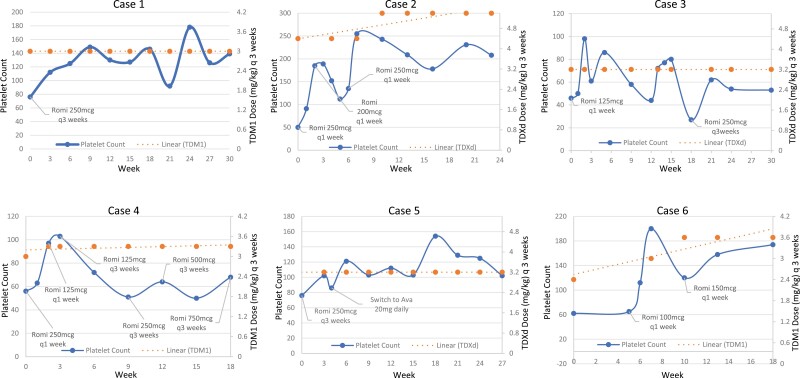
Platelet count plotted against weeks on therapy of TDM1 or TDXd. The solid blue line represents the platelet count, while the orange hashed line represents the trendline of the respective therapy and orange points are the interval at which T-DM1 or T-DXd were administered. Growth factor was initiated on marked time points on the abscissa, platelet counts at initiation of growth factor is shown on ordinate and week 0 on abscissa. TDM1: trastuzumab emtansine. TDXd, trastuzumab deruxtecan; Romi, romiplostim; Ava, avatrombopag; q3 weeks, every 3 weeks; mcg, micrograms; mg, milligram; kg, kilogram.

We identified 6 individuals who were treated with TPO-RA for thrombocytopenia associated with HER2-targeted ADC. This included 5 with metastatic disease and 1 with localized disease. Demographics, disease characteristics and breast cancer treatments are shown in Table 1. They were treated with TPO-RA, and all received romiplostim initially with 1 subsequently transitioned to avatrombopag. Thrombocytopenia was determined to be secondary to ADC therapy by hematologists due to the temporal relationship between ADC administration and the onset of thrombocytopenia, 1 individual developed an infection, but thrombocytopenia persisted post-resolution, bone marrow involvement was not formally assessed by biopsy in any of the individuals, and none received platelet transfusions. All 6 individuals were able to resume therapy. Their platelet response to TPO-RA, TPO-RA dosing as well as their dose of T-DM1 or T-DXd after initiation of TPO-RA therapy is shown in Fig. 1.

**Table 1.** Patient characteristics, treatment detail, and dose modifications for thrombocytopenia.

Age & gender	Race	Breast cancer subtype	Agent	Line of therapy	Delays	Dose reductions	Cycle of 1st reduction for TCP	Cycle of 2nd reduction for TCP	Platelet nadir	Met CIT criteria^*^
57F	White	ER-/PR-/HER2 3+	T-DM1	2nd line metastatic	0	2	6	8	52	yes
53F	White	ER-/PR-/HER2 2 + (ISH+)	T-DXd	3rd line metastatic	0	1	1	N/A	91	no^**^
36F	Asian	ER+/PR+/HER2 3+	T-DXd	4th line metastatic	3	3	5	10	24	yes
57F	White	ER+/PR-/HER2 3+	T-DM1	2nd line metastatic	0	1	18	N/A	50	yes
58F	White	ER-/PR-/HER2 3+	T-DXd	3rd line metastatic	1	2	7	21	57	yes
80F	White	ER-/PR-/HER2 3+	T-DM1	Adjuvant	2	2	4	6	50	yes

^*^Chemotherapy-induced thrombocytopenia (CIT) criteria: platelet count < 100 000/mcL for ≥ 3 weeks following last antibody-drug conjugate administration.

^**^1 individual did not meet CIT criteria while on T-DXd; however, the individual had disease progression on dose-reduced and dose-delayed T-DM1, and the reason for therapy delays and reductions was thrombocytopenia and the clinician made decision to dose-reduce the next line of therapy, T-DXd, as a result.

Abbreviations: TCP, thrombocytopenia; F, female; ER, estrogen receptor; PR, progesterone receptor; HER2, human epidermal growth factor receptor 2; 2+, 2 + by immunohistochemistry; 3+, 3 + by immunohistochemistry; ISH, in situ hybridization; T-DM1, trastuzumab emtansine; T-DXd, trastuzumab deruxtecan.

## Discussion

The 6 cases reported here demonstrate the efficacy of TPO-RA in alleviating thrombocytopenia caused by T-DXd and T-DM1. Although the thrombocytopenia in our cohort was not severe enough to cause bleeding, thrombocytopenia can be a cause of therapy discontinuation or dose reduction, or treatment delays as was the case in these individuals. Clinician’s concern that the individuals may eventually need to discontinue therapy secondary to thrombocytopenia was the reason for referral to hematology. TPO-RA use is off-label for the purpose of therapy-induced thrombocytopenia as there is no FDA-approved indication; however, it has been shown to be well tolerated in the setting of chemotherapy-induced thrombocytopenia in individuals with solid tumor malignancies without an increased risk of thrombosis, which is the primary theoretical concern when utilizing these agents.^9^ Our results show that the concomitant use of TPO-RA can help maintain dose intensity and prolong the duration of therapy in individuals treated with these HER2-targeted ADC.

These ADCs have growing indications in breast, lung, and gastric cancer.^2-8^Furthermore, these agents are well tolerated and responses are durable as seen in the DESTINY-Breast02 study of 406 individuals in which the median progression free survival (PFS) was 17.8 months in the T-DXd arm and in the EMILIA and TH3RESA studies the median PFS on T-DM1 were 9.6 months and 6.2 months, respectively.^2,10,11^ Studies have observed a higher frequency of thrombocytopenia in individuals of Asian ancestry on therapy with T-DM1 with the incidence of grade ≥ 3 thrombocytopenia as high as 0.20 in Asians.^12,13^ Among the 6 cases reported here, 2 have been on T-DM1 for over 18 months and another 2 have been on T-DXd for over 18 months, all with disease control. A study of 893 participants on T-DM1 for advanced breast cancer did not find a significant impact on PFS or overall survival for dose adjustments within 4 months of therapy initiation; however, the impact of therapy delays and dose-reductions in these agents, beyond 4 months on T-DM1 for breast cancer, and T-DXd are not well studied but there is a concern for the loss of efficacy with a deviation from the dose and schedule utilized in the registration trials.

The mechanism of thrombocytopenia in the case of T-DM1 has been proposed to be secondary to selective toxicity to megakaryocytes,^14^ without clear evidence of toxicity to hematopoietic stem cells which is the proposed mechanism of conventional chemotherapy-induced thrombocytopenia.^15^ In vitro and murine studies suggest that megakaryocytes internalize T-DM1 either via FcγRIIA or by micropinocytosis and the release of DM1 intracellularly leads to impairment of megakaryocyte differentiation and proplatelet formation.^8,10^ Since the production of endogenous thrombopoietin is constant regardless of platelet count, TPO-RA by stimulating megakaryocyte growth, differentiation, and platelet production appears to be able to overcome the selective toxicity of HER2-targeted ADC to megakaryocytes.

Dosing of romiplostim varied among our cohort. The optimal dosing interval to support chemotherapy-induced thrombocytopenia remains uncertain for romiplostim. The median dose in the immune thrombocytopenia studies is 2-3 mcg/kg. The starting dose and interval varied in our patients and is shown in Fig. 1 and dose was titrated based on patient response. Our data suggest that a 3-weekly schedule of a fixed dose of romiplostim is feasible in this setting, and we suggest titrating dose to maintain a platelet count above 50 000/µL. We did not observe any unexpected adverse events attributable to TPO-RA, and no venous thromboembolism events were observed.

Although TPO-RA has shown limited efficacy to alleviate thrombocytopenia induced by conventional chemotherapy, they seem to be efficacious in the setting of HER2-targeted ADC therapy based on our small series. To our knowledge, this is the first report of the successful utilization of TPO-RA for thrombocytopenia secondary to T-DM1 and T-DXd therapy. Our study is limited by a small sample size, but we find the results encouraging. The role of TPO-RA in supporting the use of currently approved HER2-targeted ADC warrants further investigation in prospective trials.

## Data Availability

The data underlying this article will be shared on reasonable request to the corresponding author.
